# A Novel Approach to Eliminate the Effect of External Stress on Interdiffusivity Measurement

**DOI:** 10.3390/ma10080961

**Published:** 2017-08-17

**Authors:** Weimin Chen, Qin Li, Lijun Zhang

**Affiliations:** 1 Institute of Advanced Wear & Corrosion Resistant and Functional Materials, Jinan University, Guangzhou 510632, Guangdong, China; chenweiming126@163.com or chenweiming126@jnu.edu.cn; 2 State Key Laboratory of Powder Metallurgy, Central South University, Changsha 410083, Hunan, China; qinli333@csu.edu.cn

**Keywords:** interdiffusivity, Co–Ni alloy, diffusion, diffusion couple, pragmatic numerical inverse method

## Abstract

In this paper, the interdiffusivities in fcc Co–Ni alloys at 1373 K due to different types of diffusion couple experiments were firstly re-calculated via the unified Wagner method based on the measured composition profiles. Their maximum difference due to different approaches for diffusion couple preparation was found to be larger than one order of magnitude. Then, a comprehensive analysis on the effect of different preparation methods was performed. After that, a two-step diffusion couple technique in combination with the pragmatic numerical inverse method was proposed to determine the accurate interdiffusivities by eliminating the effect of external stress. Such a novel approach was successfully applied in the binary fcc Co–Ni alloys for demonstration purposes. Moreover, it is anticipated that such novel approach can be utilized as the standard method for accurate interdiffusivity measurement, and the resultant accurate interdiffusivities in different alloys may serve as a benchmark for the later experimental and theoretical studies.

## 1. Introduction

Diffusion is an omnipresent but important phenomenon in various disciplines and applications in physical [1], chemical [2,3,4,5], biological [6], geologic [7], materials science and engineering processes [8,9]. Dr. Fick, a renowned physiologist of the 19th century, developed the mathematical framework to describe the phenomenon of diffusion, which subsequently became known as Fick’s laws [10]. Fick’s laws improve and quantify our understanding of diffusion-related phenomena and serve as an important theoretical basis for phase transition modeling and simulations [11,12]. In order to obtain quantitative descriptions of diffusion kinetics using Fick’s laws, one of the most important aspects is the input of reliable interdiffusivities, which are usually pressure-, temperature- and composition-dependent quantities [13,14,15].

The diffusion couple (or diffusion multiple) technique is the most frequently used method to determine the interdiffusion coefficients and/or kinetic coefficients in the gas, liquid and solid states [16,17,18,19]. Literature surveys [20,21,22,23] indicate that most of the related publications available in the literature focus on the measurement of interdiffusivities in a single phase of the binary or ternary alloys with the aid of several reliable calculation methods (i.e., the Boltzmann-Matano method for binary systems [24], or the newly developed pragmatic numerical inverse method for binary, ternary and multicomponent systems [25,26]) together with the scientific error propagation method [27,28]. It should be noted that the so-called pragmatic numerical inverse method was augmented by fully considering the variations of interdiffusion flux into the calculation procedures [26]. Moreover, the accuracy of this augmented numerical inverse method was carefully verified in different alloys, ranging from binary, ternary to multicomponent systems. The results from the numerical inverse method for the binary and ternary systems were confirmed to be consistent with those from the traditional methods [26].

**Figure 1:**
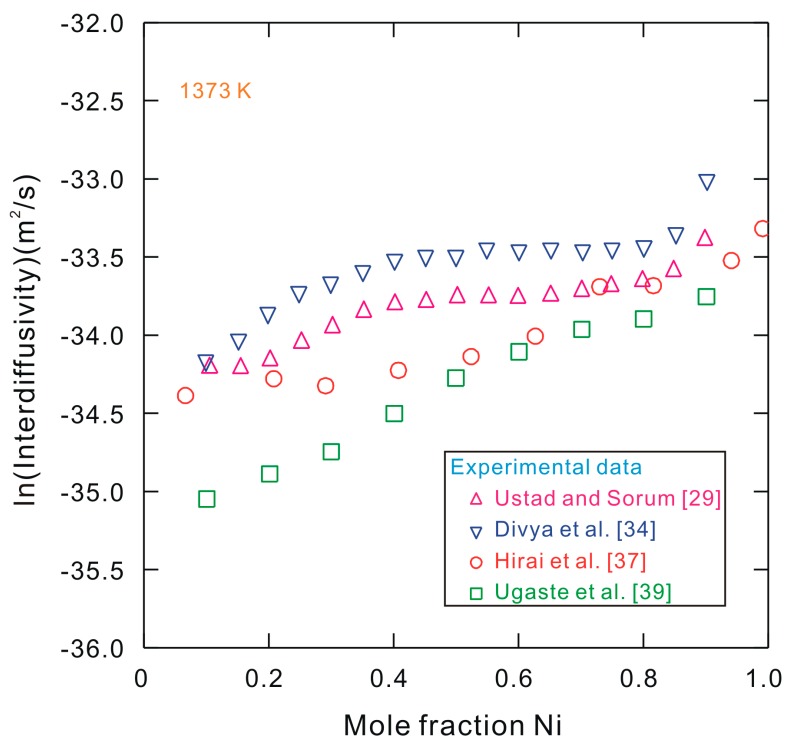
Natural logarithm of interdiffusivities in fcc Co–Ni system at 1373 K, reported from the literature [29,34,37,39].

Ideally, interdiffusion initiates, in principle, once the two couples contact each other. However, it is very difficult to achieve the good contact interface during actual preparation of diffusion couples/multiples. In general, there are two types of techniques for the preparation of diffusion couples with a good contact interface. One is the pre-contacted diffusion couple technique, while the other is the pressing diffusion couple technique. For the pre-contacted diffusion couple technique, e.g., by using electroplating [29], tiny thickness of the deposit and the small grain size exist after pre-treatment, which raises the effect of the grain boundary diffusivities, and thus affects the determined interdiffusivities. Thus, the precise measurement of “true” interdiffusivities is very difficult in this case. While for the pressing diffusion couple techniques, several approaches are often implemented, such as by engaging a special fixture [30,31,32,33,34,35,36], adopting a friction welding technique [37], using a uniaxial hot-pressing technique [38,39,40], or by invoking a hot isostatic pressing (HIP) technique [41]. However, due to the narrow contact area between the two end–members, the applied stress on the samples cannot be neglected as “zero”, and thus it also affects the determined interdiffusivities. The above two types of experimental techniques have been utilized to investigate the interdiffusion behavior of alloys, e.g., the face-centered cubic (fcc) solid phase of the Co–Ni system [29,34,37,39,41]. Figure 1 shows the interdiffusivities of the binary Co–Ni system at 1373 K presented in the literature [29,34,37,39]. It can be observed that the maximum spread among these experimental interdiffusivities from four research groups is about 180%. Therefore, such diverse interdiffusivities cannot serve as the standard data adopted in subsequent phase transition simulations, or for the validation of theoretical predictions like first-principle (FP) calculations and molecular dynamic (MD) simulations. In fact, fcc Co–Ni systems are not an extreme case, and similar phenomena also occur in other systems, like binary Ni–Pt and Co–Pt systems [42]. Therefore, there is an urgent need to develop an approach with which the effect of external stress caused by different preparation methods of diffusion couples on the determined interdiffusivities can be disregarded. Some other factors, like the purities for raw materials, the uncertainty in experimental equipment, and the uncertainty of determining the exact annealing time, may also contribute to experimental uncertainties. However, these fall outside the focus in this work, and are not discussed here.

Consequently, the major purpose of the present work is to obtain the interdiffusivities using the diffusion couple technique in the absence of external stress. In order to eliminate the effect of external stress during the interdiffusivity measurement using the pressing diffusion couple techniques, a well-designed two-step diffusion couple technique together with the pragmatic numerical inverse method is to be developed in this work. The binary fcc Co–Ni system is thus selected as the target for demonstrating the methodology here due to (i) its technical importance in Co–based and Ni–based superalloys and high-temperature shape memory alloys [43,44]; and (ii) the reported interdiffusion information using various techniques at 1373 K [34,37,41] can be used for a comprehensive comparison.

The main research tasks in the present paper are (i) to utilize the unified Wagner method [45] for re-determination of the interdiffusivities in the Co–Ni system at 1373 K from the experimental composition profiles obtained by using the friction welding method [37] and the HIP technique [41]; (ii) to perform the solid/solid Co/Ni diffusion couple experiments by using both a Mo jig and the uniaxial hot-pressing technique at 1373 K for 2 h and determine the corresponding interdiffusivities using the unified Wagner method [45] for a direct comparison with the literature data; and (iii) to conduct a stress-free annealing treatment of diffusion couples at 1373 K for 98 h after uniaxial hot-pressing and utilize the pragmatic numerical inverse method [26] to determine the interdiffusivities during the annealing period in the absence of external stress.

## 2. Materials and Methods

### 2.1. Experimental Procedure

Cobalt (1 mm thick, purity: 99.95 wt·%) and nickel (1 mm thick, purity: 99.5 wt·%) foils purchased from Alfa Aesar (Shanghai, China) Chemicals Co., Ltd. were used as starting materials, and arc-melted into pure Co and Ni buttons in a high purity argon atmosphere using an arc melting furnace (WKDHL-1, Optoelectronics Co., Ltd., Beijing, China), which was equipped with a nonconsumable tungsten electrode and a water-cooled copper anode. The buttons were linearly cut into blocks with the size of 5 × 5 × 1 mm^3^. These blocks were ground by using SiC papers (120, 600, 1000, 1500 and 2000 grit), then sealed into evacuated quartz tubes and homogenized at 1473 ± 2 K for 14 days. All the samples were ground on SiC paper to remove surface contamination. After being polished, one diffusion couple was assembled by using a special Mo jig while three other diffusion couples were prepared by a self-assembly vacuum hot-pressing apparatus with different stresses (i.e., 5600 Pa, 10 and 20 MPa). All the diffusion couples were annealed at 1373 ± 3 K for 2 h. Moreover, three diffusion assemblies after hot-pressing annealing at 1373 ± 3 K for 2 h were sealed into evacuated quartz tubes again, and then annealed at 1373 ± 3 K for 98 h. After standard metallographic techniques, the composition profiles of each diffusion couple were determined by using field emission electron probe micro analysis (Fe-EPMA, JXA-8530, JEOL, Tokyo, Japan) with an accelerating voltage of 15 kV. Here, the pure Ni (purity: 99.95 wt·%; source: Alfa Aesar (China) Chemicals Co., Ltd.) and Co (purity: 99.5 wt·%; source: Alfa Aesar (China) Chemicals Co., Ltd.) at the terminal ends of the diffusion couples were used as calibration standards and the Ni–Lα (LiF, Lithium fluoride) and Co–Lα (LiF, Lithium fluoride) X-ray peaks were used for quantification. Variations in alloy compositions were determined to be within ±0.5 at·% for each component.

### 2.2. Wagner Method for Binary Interdiffusivity Calculation

In binary diffusion couples, the interdiffusion flux *J* at any location *x*′ can be calculated directly from the normalized composition profile [45],

(1) $$J=\frac{\left(N^{L}-N^{R})\cdot V_{m}(x\prime \right)}{2t}\{[1-Y(x\prime )]\cdot \int_{x^{L}}^{x\prime }\frac{Y(x)}{V_{m}(x)}dx+Y(x\prime )\cdot \int_{x\prime }^{x^{R}}[\frac{1-Y(x)}{V_{m}(x)}]dx\}$$

where *t* is the diffusion time, *V_m_*(*x*) is the molar volume at any location *x*, and *N^L^* and *N^R^* are the composition normalized variables at the far left end (*x^L^*) and far right end (*x^R^*) of the diffusion couple, respectively. According to a recent report [46], the molar volume *V_m_* of the Co–Ni alloys can be expressed by the linear relation (*V_m_* = *N*_Co _× *V*_Co _+ *N*_Ni _× *V*_Ni_), which has a minor effect on the calculations of the interdiffusion flux. Here, the molar volumes for pure Ni (*V*_Ni_) and Co (*V*_Co_) were set to be 6.6 × 10^−6^ m^3^/mol and 6.7 × 10^−6^ m^3^/mol [35], respectively. A composition ratio *Y* at the distance coordinate *x*’ is defined by,

(2) $$Y(x\prime )=\frac{N(x\prime )-N^{L}}{N^{R}-N^{L}}$$

On the basis of Fick’s first law [47], the composition–dependent interdiffusivities $\tilde{D}$ can be calculated by using the following expression [45],

(3) $$\tilde{D}=\frac{\left(N^{R}-N^{L})\cdot V_{m}(x\prime \right)}{2t\cdot (\partial N/\partial x){|}_{x\prime }}\{[1-Y(x\prime )]\cdot \int_{x^{L}}^{x\prime }\frac{Y(x)}{V_{m}(x)}dx+Y(x\prime )\cdot \int_{x\prime }^{x^{R}}[\frac{1-Y(x)}{V_{m}(x)}]dx\}$$

It is noted that the so-called “interdiffusivity” here is actually the mole fraction interdiffusivity [48].

### 2.3. Numerical Inverse Method for Binary Interdiffusivity Calculation

Very recently, a numerical inverse method for determining the composition-dependent interdiffusivities in alloys from a single diffusion couple was newly proposed by the present authors [25,26], and can also be directly applied to the binary system. In order to assign more physical meanings, the mobility for element *i* is given by,

(4) $$M_{i}=\frac{1}{RT}\exp(\frac{N_{A}\Delta G_{i}^{A}+N_{B}\Delta G_{i}^{B}+N_{A}N_{B}\Delta G_{i}^{A,B}}{RT})$$

where *R* is the gas constant, *T* is absolute temperature, $\Delta G_{i}^{A}$ and $\Delta G_{i}^{B}$ are the end-members for diffusion of element *i* in element *A* and *B*, while $\Delta G_{i}^{A,B}$ is the interaction parameter for the mobility of element *i* in the *A*–*B* binary system. Here, four end-members for fcc Co–Ni system ($\Delta G_{Co}^{Co}$, $\Delta G_{Co}^{Ni}$, $\Delta G_{Ni}^{Ni}$ and $\Delta G_{Ni}^{Co}$) are fixed by the assessed parameters [33,41,49]. As the adjustable parameter, the coefficient $\Delta G_{i}^{A,B}$ needs to be evaluated on the basis of one or several sets of composition profiles. In addition, the interdiffusivities and the mobility *M_i_* are related by [50,51],

(5) $$\tilde{D}=RT(N_{A}M_{B}+N_{B}M_{A})\Phi [1+s\frac{2N_{A}N_{B}{\left(M_{A}-M_{B}\right)}^{2}}{A_{0}(N_{A}M_{B}+N_{B}M_{A})(N_{A}M_{A}+N_{B}M_{B})}]$$

The vacancy-wind effect will be considered if *s* equals 1, while it will not be considered if *s* equals 0. The parameter *A*_0_ is a factor depending only on crystal structure (e.g., 7.15 for fcc crystals) [50]. The thermodynamic factor Φ is expressed by,

(6) $$\Phi =\frac{N_{A}N_{B}}{RT}\cdot \frac{d^{2}G}{dN^{2}}$$

where the Gibbs energy *G* can be obtained from the corresponding thermodynamic descriptions for fcc Co–Ni system [52]. The composition evolution of component *i* in the binary *A*–*B* system can be governed by Fick’s second law,

(7) $$\frac{\partial N_{i}}{\partial t}=\frac{\partial }{\partial x}(\tilde{D}\frac{\partial N_{i}}{\partial x})$$

By combining Equations (4)–(7), the composition profiles of components *A* and *B* for the given coefficients $\Delta G_{A}^{A,B}$ and $\Delta G_{B}^{A,B}$ can be calculated. An optimal set of adjustable parameters, such as $\Delta G_{A}^{A,B}$ and $\Delta G_{B}^{A,B}$, were carefully chosen by iterative fitting until the minimization of the error between the measured and calculated composition profiles is achieved,

(8) $$\min<error>=\min<w_{c}\cdot \sum_{j=1}^{N}\frac{\left(\left|c_{j}^{\mathrm{cal}}-c_{j}^{\exp}\right|\right)}{c_{j}^{\exp}}+w_{J}\cdot \sum_{j=1}^{N}\frac{\left(\left|{\tilde{J}}_{j}^{\mathrm{cal}}-{\tilde{J}}_{j}^{\exp}\right|\right)}{\left|{\tilde{J}}_{j}^{\exp}\right|}>$$

where $c_{j}^{\mathrm{cal}}$, $c_{j}^{\exp}$, ${\tilde{J}}_{j}^{\mathrm{cal}}$ and ${\tilde{J}}_{j}^{\exp}$ are the calculated and the experimental composition and interdiffusion flux of component *A* or *B* at the *j*th point, respectively, and *N* is the number of the experimental data. In addition, the weights of composition and interdiffusion flux in the minimization are determined by the divergence of the experimental data. With the optimal set of fitting parameters, the composition profiles can be obtained, and the composition-dependent interdiffusivites in the target binary system can be computed via Equations (5)–(7). The standard deviation (SD) for both Wagner method and numerical inverse method can be calculated by using the scientific statistical method [27,28].

## 3. Results and Discussion

**Figure 2:**
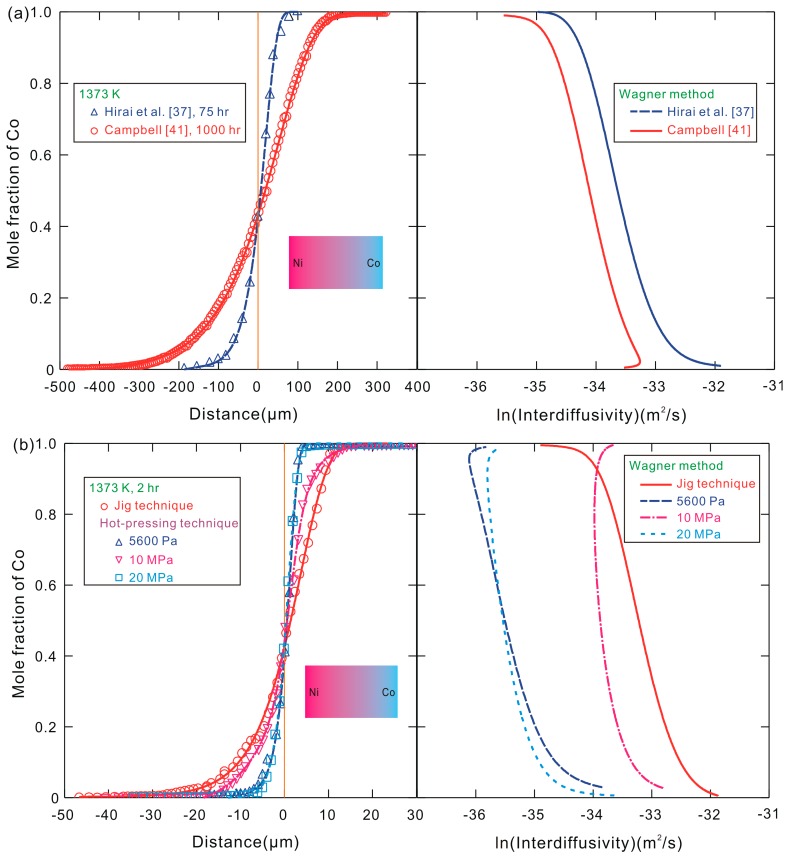
Composition profiles and natural logarithm of the corresponding interdiffusivities for Co–Ni diffusion couples obtained (**a**) from the literature (Note: re-calculated using the unified Wagner method); and (**b**) from the present experiments. Symbols designate the results from the prior experimental measurements in Refs. [37,41] and the present experiments while solid and dash lines are the fitted composition profile and the interdiffusivities calculated by using the Wagner method based on the literature data [37,41] and the present results.

The experimental composition profiles illustrated in References [37,41] can be well reproduced by the fitted curves as shown in Figure 2a. Here, the “zero” point of the distance for both of the experimental data sets is shifted to the Matano plane position *x*_M_, which is defined by [24] as

(9) $$\int_{N^{L}}^{N^{R}}(x-x_{M})dN=0$$

Based on the fitted data, the interdiffusivities over a wide range of compositions for the binary fcc Co–Ni system at 1373 K can be further calculated by using the Wagner method [45], and the corresponding results are shown in Figure 2a. It can be seen that the calculated interdiffusivities based on the experimental data [37] are approximately 60% higher than those from the measured results [41].

Figure 2b shows the composition profiles of fcc Co–Ni diffusion couples obtained from the present experiments performed by means of a vacuum hot-pressing apparatus and a special Mo jig at a temperature of 1373 K for 2 h. The corresponding binary interdiffusivities for four sets of the measured data are calculated by the Wagner method [45] and the corresponding results are presented in Figure 2b. It can be observed that the interdiffusivities of the diffusion couple prepared by using the special jig are about 2 times larger than the data obtained with an applied stress of 10 MPa, and about 10 times larger than the results obtained under the uniaxial stresses of 5600 Pa and 20 MPa. The detailed issue about the effect of the uniaxial stress on the interdiffusion might be caused by the formation of vacancy and the atomic migration in the elastic deformation state, which is not within the scope of the present work, and will be subject to further detailed investigation.

**Figure 3:**
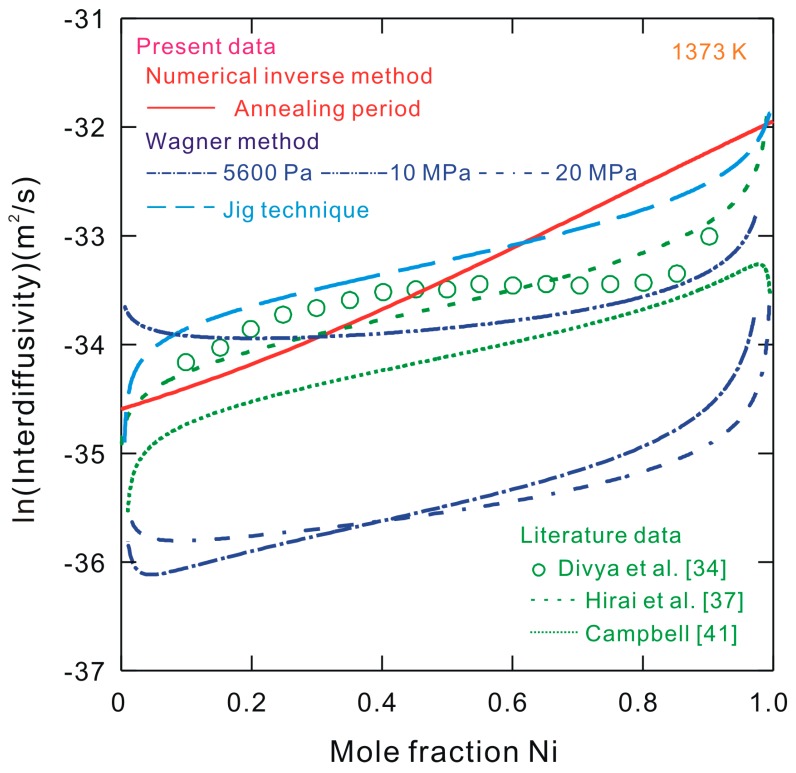
Natural logarithm of the interdiffusivities obtained by using the Wagner method and the pragmatic numerical inverse method in the binary Co–Ni system at 1373 K, compared with the literature data [34,37,41].

Figure 3 shows the natural logarithm (ln) of the present interdiffusivities calculated by using the Wagner method in a Co–Ni system at 1373 K, compared with the data obtained from the literature [34,37,41] (Note: these were also re-calculated by the Wagner method in order to retain consistency). Because the same calculation method was adopted, the error introduced by the different calculation methods can be regarded as negligible. As can be seen in the figure, the interdiffusivities obtained in this study by using the jig technique are 40%, 100% and 200% larger than the data reported by Divya et al. [34], Hirai et al. [37] and Campbell [41], respectively. This fact suggests that with such a special fixture/jig, a uniaxial pressure should be applied to the end members of the prepared diffusion couples. The introduced inexact stresses may result in 40–100% difference of the obtained binary interdiffusivities. Moreover, it can also be seen in Figure 3 that the interdiffusivities under a stress of 10 MPa are approximately 5.5 times larger than those recorded under the stresses of 5600 Pa and 20 MPa. This comparison also indicates that the interdiffusion behavior is definitely affected by the uniaxial stress applied on the samples.

**Figure 4:**
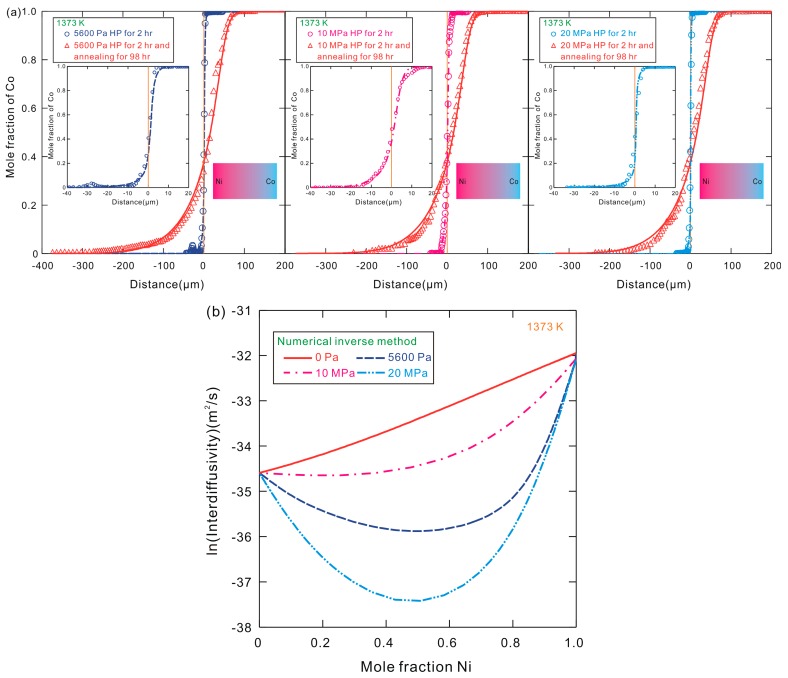
(**a**) Composition profiles and (**b**) natural logarithm of the corresponding interdiffusivities for Co–Ni diffusion couples after hot-pressing at 1373 K for 2 h under different stresses and annealing in a quartz capsule at 1373 K for 98 h. Symbols designate the data from the measurements in the present work while solid and dash lines are the simulated data from the pragmatic numerical inverse method.

As already stated in Section 2, the three diffusion couples after the hot-pressing for 2 h were further annealed in a quartz capsule at 1373 K for 98 h. Such diffusion couples after the hot-pressing period (Note: the sample was under external stress and kept in the elastic regime during this period) and the annealing period (Note: the sample was under no external stress during this period) can provide two sets of the composition profiles after two periods. Then, the accurate interdiffusivities, without the effect of external stress, can be determined by using the proposed pragmatic numerical inverse method [26]. The detailed procedure for the calculation of accurate interdiffusivities is described as follows: firstly, the composition profiles of the diffusion couples after hot-pressing for 2 h were simulated by using the numerical inverse method with an excellent fit to the experimental data measured in the first step (i.e., the hot-pressing period); secondly, the diffusion couples after hot-pressing were annealed with no external stress for 98 h, the simulated composition profiles recorded in the first step were set as the initial input to perform the interdiffusion simulation for the subsequent annealing period, and the new adjustable parameters during the annealing period were then obtained from the numerical inverse method to reproduce the experimental composition profiles measured in the second step (i.e., the hot-pressing period plus the annealing period); thirdly, the binary composition-dependent interdiffusivities in fcc Co–Ni system during the two periods could be completely separated and extracted on the basis of Equations (4) and (5) from different sets of the adjustable parameters during the two periods of hot-pressing and annealing. Figure 4 shows the composition profiles and the corresponding interdiffusivities obtained by using the two-step diffusion couple technique in combination with the numerical inverse method [26]. Symbols designate the measured data in the present work while dash lines represent the simulated data from the pragmatic numerical inverse method after the hot-pressing period. The solid lines in the composition-distance plots are the simulated composition profiles by using the pragmatic numerical inverse method after both the hot-pressing period and the annealing period, while the solid line in the composition-interdiffusivity plot represents the interdiffusivity during the annealing period (i.e., under no external stress). It should be noted that the same interdiffusivity data without the external stress can be obtained from the experimental data of three two-step diffusion couples under different stresses. The measured composition profiles of fcc Co–Ni diffusion couples annealed for 2 h and 100 h were well reproduced by the simulated values, indicating that the present interdiffusivities obtained by the numerical inverse method are reliable. Moreover, the difference between the corresponding interdiffusivities during the two periods of hot-pressing and annealing may be due to (i) the relatively slow migration rate of atoms before the good contact between the couples in the initial stage during the hot-pressing period; and (ii) the existence of external uniaxial stress during the hot-pressing period while not during the annealing period. Under these conditions, the interdiffusivities obtained during the annealing period without the external uniaxial stress can be considered as the accurate interdiffusivities of fcc Co–Ni alloys if other factors, such as the purity of raw materials and the uncertainty of experimental equipments and the exact annealing time, are disregarded.

Figure 3 presents the natural logarithm of the accurate interdiffusivities (i.e., under no applied stress during the annealing period) obtained using the pragmatic numerical inverse method for the Co–Ni system at 1373 K, compared with those calculated using the unified Wagner method based on the composition profiles from the present work and the literature [34,37,41]. The accurate binary interdiffusivities in Co-rich region are slightly lower than the results from the literature [34,37] and the present jig technique, while those in the Ni-rich region are slightly greater than the data obtained in Refs. [34,37] and from the present jig experiment. It can be deduced that the stress introduced by using the jig technique has a definite influence on the interdiffusivities in alloys. Figure 3 also shows that the accurate interdiffusivities at 1373 K are 50–200% larger than the literature data obtained with the aid of the HIP technique [41], which reveals that the interdiffusion behavior in the Co–Ni system [41] may be markedly influenced by the high Ar pressure during the implementation of the HIP period [53]. Good interfacial contacts of diffusion multiple are achieved by plastic deformation induced by HIP-can material at a certain temperature under high argon pressure [54]. However, the effect of plastic deformation on the interdiffusion behavior is very complex. An explanation for this is related to the creep behavior of the materials involved. It is noted that the interdiffusion behaviors under the plastic deformation may be different from the behavior observed in the regime of elastic deformation.

Actually, a similar two-step diffusion technique was also utilized by previous researchers [41,54], but no measurements were performed during the first step. Instead, the annealing time was extended longer than the HIP time in order to reduce the effect of the preparation process of the diffusion couples. However, this treatment process cannot eliminate the effect of the initial preparation techniques. Moreover, van Loo and Rieck [55] also reported the existence of an incubation stage during the growth of the intermetallic compounds (IMCs). Therefore, Yuan et al. [56] introduced an effective diffusion time to eliminate the effect of the initial transient stage. With such a similar two-step diffusion stage, Yuan et al. [56] obtained the parabolic growth constants of the IMCs. It should be kept in mind that the effective diffusion time is corrected by truncating the incubation time of the early starting stage, which is only suitable for the calculation of the growth constant, and not for the interdiffusivity calculation. This is the reason why the traditional Wagner method was adopted in an effort to determine the binary interdiffusivity by Yuan and her coworkers [56]. In their study, the effect of the initial transient stage was still included. In addition, the effects of stress on material structure and coatings has been pointed out in the literature [57,58]. In the present work, a two-step diffusion couple technique together with the numerical inverse method [25,26] was proposed as a more appropriate approach for obtaining two sets of the interdiffusivities during the two different periods. The interdiffusivities during the first period were determined following the standard method previously described in References [25,26]. The composition profiles after the first period were recorded and adopted as the initial composition distributions for the second step. This implies that the interdiffusion fluxes existed before the beginning of the second step. Then, the interdiffusivities in the second step can be independently obtained, which may not be close to those in the first step. Due to the fact that the diffusion couples in the second step were annealed without external stress, the interdiffusivities during the second period can be considered to be the accurate interdiffusivities without any external stress if other factors can be neglected.

## 4. Conclusions

- We pointed out the noticeable influence of different diffusion couple preparation approaches on the determined interdiffusivities, which was neglected in numerous previous studies. Based on the measured composition profiles of binary fcc Co/Ni diffusion couples prepared by different experimental methods, the unified Wagner method was adopted for a quantitative analysis of the effect of different preparation methods.
- We proposed a two-step diffusion couple technique combined with the pragmatic numerical inverse method for obtaining the accurate interdiffusivities without any external stress, and it was successfully demonstrated in binary fcc Co–Ni alloys. Moreover, it should be noted that the variations of composition profiles and/or interdiffusion flux obtained in the first step are also considered in the determination of the interdiffusivity in the second step, which is always neglected prior to this work.
- The proposed novel approach is anticipated to be a generally applicable approach for other binary and multicomponent systems, and the resultant accurate interdiffusivities without any external stress may serve as a benchmark for future experimental and theoretical studies.
